# Supplementary material for: A systematic review assessing the quality of patient reported outcomes measures in dry eye diseases
Source: PLoS One. 2021 Aug 9;16(8):e0253857. doi: 10.1371/journal.pone.0253857 (PMC8351938; doi:10.1371/journal.pone.0253857)
Supplement: S1 Fig — (DOCX) [file pone.0253857.s001.docx]

**Figure 1 - Systematic Review PRISMA 2009 Flow Diagram**

Number of records identified (n=5761) via:

- MEDLINE (Ovid) (n=1,710)
- EMBASE (n= 3,556)
- PsycINFO (n=495)
- CINAHL+ (n= 0)
- Cochrane Library (n= 0)


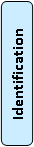


Number of records excluded (n=5061), reasons:

- Out of scope (n=3,391)
- No mention of PROMs (n= 1,215)
- Other (n= 455)


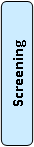


Number of title/abstract records screened after 573 duplicates removed (n=5,188)

Number of papers excluded (n=118), reasons:

- Out of scope (n=113)
- Systematic Reviews (n=2)
- Abstracts (n=1)
- Clinician Tools (n=2)


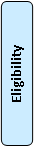


Number of full-text records assessed for eligibility (n=127)

Number of additional records included following hand-search of reference lists and citation searches (n=8)


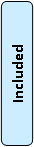


Final number of full-text articles included **(n=17)**
