# Supplementary material for: A systematic review assessing the quality of patient reported outcomes measures in dry eye diseases
Source: PLoS One. 2021 Aug 9;16(8):e0253857. doi: 10.1371/journal.pone.0253857 (PMC8351938; doi:10.1371/journal.pone.0253857)
Supplement: S1 Table — (DOCX) [file pone.0253857.s002.docx]

Supplementary Table 1 COSMIN definitions of domains, measurement properties, and aspects of measurement properties

| **Term** | | | **Definition** |
| --- | --- | --- | --- |
| **Domain** | **Measurement property** | **Aspect of a measurement**  **property** |  |
| Reliability |  |  | The degree to which the measurement is free from  measurement error |
| Reliability  (extended definition) |  |  | The extent to which scores for patients who have not changed are the same for repeated measurement under several conditions: e.g. using different sets of items from the same PROM (internal consistency); over time (test‐retest); by different persons on the same occasion (inter‐ rater); or by the same persons (i.e. raters or responders) on  different occasions (intra‐rater) |
|  | Internal consistency |  | The degree of the interrelatedness among the items |
|  | Reliability |  | The proportion of the total variance in the measurements which is due to ‘true’† differences  between patients |
|  | Measurement error |  | The systematic and random error of a patient’s score that is not attributed to true changes in the  construct to be measured |
| Validity |  |  | The degree to which a PROM  measures the construct(s) it purports to measure |
|  | Content validity |  | The degree to which the content of a PROM is an adequate  reflection of the construct to be measured |
|  |  | Face validity | The degree to which (the items of) a PROM indeed looks as though they are an adequate  reflection of the construct to be measured |
