# Supplementary material for: A systematic review assessing the quality of patient reported outcomes measures in dry eye diseases
Source: PLoS One. 2021 Aug 9;16(8):e0253857. doi: 10.1371/journal.pone.0253857 (PMC8351938; doi:10.1371/journal.pone.0253857)
Supplement: S2 Table — (DOCX) [file pone.0253857.s003.docx]

Supplementary Table 2 Summary of FDA PRO requirements

|  | Identify concepts and develop conceptual framework | - Identify concept and domains that are important to patients - Determine intended population and research application - Hypothesize expected relationships among concepts |
| --- | --- | --- |
|  | Create instrument | - Generate items - Choose administration methods, recall period and response scales - Draft instructions - Format instrument - Draft procedures for scoring and administration - Pilot test draft instrument - Refine instrument and procedures |
|  | Assess measurement properties | - Assess score reliability, validity and ability to detect change - Evaluate administrative and respondent burden - Add, delete, or revise items - Identify meaningful differences in scores - Finalize instrument formats, scoring procedures and training materials |
|  | Modify instrument | - Change concept measured, population studies, research application, instrumentation or method of administration |
