# Supplementary material for: A systematic review assessing the quality of patient reported outcomes measures in dry eye diseases
Source: PLoS One. 2021 Aug 9;16(8):e0253857. doi: 10.1371/journal.pone.0253857 (PMC8351938; doi:10.1371/journal.pone.0253857)
Supplement: S3 Table — (DOCX) [file pone.0253857.s004.docx]

**S3 Table. Search strategy proms in dry eye disease and ocular surface disease**

**SEARCH STRATEGY PROMS IN DRY EYE DISEASE AND OCULAR SURFACE DISEASE**

- Search strategy applied:
  1. Name of the electronic databases used for searching including the interface used: MEDLINE (Ovid) and EMBASE (Ovid) were used. Additionally, PsycINFO (Ovid) and CINAHL Plus (EBSCO) were searched.
  2. Filters applied for MEDLINE, EMBASE, PsycINFO and CINAHL Plus version: See below
- Definition of construct. All search terms used:
  1. ocular surface disease or OSD
  2. ocular surface condition
  3. ocular surface disturbance
  4. ocular surface syndrome
  5. dry eye disease or DED
  6. dry eye condition
  7. dry eye disturbance
  8. dry eye syndrome or DES
  9. dry eye or DE
  10. dry eyes
  11. keratoconjunctivitis sicca or KCS
  12. keratitis sicca or KS
  13. dysfunctional tear syndrome
  14. lacrimal keratoconjunctivitis
  15. evaporative tear deficiency
  16. aqueous tear deficiency
  17. lacrimal deficiency
  18. tear deficiency
  19. tear evaporation
  20. depleted tear secretion
  21. non-Sjögren keratoconjunctivitis sicca
  22. Sjögren’s Syndrome
  23. Sjögren’s syndrome-related dry eye disease
  24. Sjögren’s syndrome-related ocular surface disease
- Restrictions:
  1. human studies only

**Logbook search:**

1. MEDLINE (Ovid):, restrictions to: humans only

| **MEDLINE (Ovid)** | |
| --- | --- |
| Content/construct | Nr of citations |
| 1. (ocular adj surface adj disease*) | 596 |
| 1. (OSD) | 67 |
| 1. (ocular adj surface adj condition*) | 15 |
| 1. (ocular adj surface adj disturbance*) | 1 |
| 1. (ocular adj surface adj syndrome*) | 1 |
| 1. (dry adj eye adj disease*) | 462 |
| 1. (dry adj eye adj condition*) | 15 |
| 1. (dry adj eye adj disturbance*) | 0 |
| 1. (dry adj eye adj syndrome*) | 1,221 |
| 1. (dry adj eye*) | 1,686 |
| 1. (DE) | 57,790 |
| 1. (DED) | 260 |
| 1. (keratoconjunctivitis adj sicca) | 127 |
| 1. (KCS) | 140 |
| 1. (keratitis adj sicca) | 4 |
| 1. (KS) | 863 |
| 1. (dysfunctional adj tear adj syndrome*) | 3 |
| 1. (lacrimal adj keratoconjunctivitis) | 1 |
| 1. (evaporative adj tear adj deficiency) | 0 |
| 1. (aqueous adj tear adj deficiency) | 10 |
| 1. (lacrimal adj deficiency) | 1 |
| 1. (tear adj deficiency) | 17 |
| 1. (tear adj evaporation) | 28 |
| 1. (depleted adj tear adj secretion) | 0 |
| 1. (non-Sjögren adj keratoconjunctivitis adj sicca) | 0 |
| 1. (sjogren adj syndrome) | 459 |
| 1. (sjogren adj syndrome adj related adj dry adj eye adj disease) | 0 |
| 1. (sjogren adj syndrome adj related adj ocular adj surface adj disease | 0 |
| 1. (sjogren adj syndrome adj dry adj eye*) | 6 |
| **TOTAL** | 63,745 |

+ AND filter (1) applied:

(PRO integration or Clinical PRO application* or telePRO or automated PRO algorithm* or screening purpose* or PRO questionnaire* or Patient-reported outcome questionnaire* or Patient-reported symptom* or Patient-centred care or Patient self-report* or Self-report health or Self-rated health or Self-reported measure* of health or Health outcome* or Health communication* or Hospital performance evaluation* or Automated telephone survey system* or paper-based survey* or web-based survey* or web-based PRO platform* or web-based system* or PRO collection* or PRO measure* or PRO intervention* or PRO assessment intervention* or PRO data or PRO assessment* or Routine PRO assessment* or Routine PRO collection or Symptom assessment* or Symptom monitoring or Symptom data or Functional status or Electronic PRO assessment* or Electronic PRO system* or ePRO or ePRO* or ePRO system* or PRO system* or Generic PRO system* or PRO-based clinical alert system*).mp.

| **MEDLINE (Ovid) + filter (1)** | |
| --- | --- |
| Content/construct | Nr of citations |
| 1. (ocular adj surface adj disease*) | 21 |
| 1. (OSD) | 3 |
| 1. (ocular adj surface adj condition*) | 0 |
| 1. (ocular adj surface adj disturbance*) | 0 |
| 1. (ocular adj surface adj syndrome*) | 0 |
| 1. (dry adj eye adj disease*) | 21 |
| 1. (dry adj eye adj condition*) | 0 |
| 1. (dry adj eye adj disturbance*) | 0 |
| 1. (dry adj eye adj syndrome*) | 48 |
| 1. (dry adj eye*) | 58 |
| 1. (DE) | 1,498 |
| 1. (DED) | 25 |
| 1. (keratoconjunctivitis adj sicca) | 8 |
| 1. (KCS) | 5 |
| 1. (keratitis adj sicca) | 0 |
| 1. (KS) | 13 |
| 1. (dysfunctional adj tear adj syndrome*) | 0 |
| 1. (lacrimal adj keratoconjunctivitis) | 0 |
| 1. (evaporative adj tear adj deficiency) | 0 |
| 1. (aqueous adj tear adj deficiency) | 1 |
| 1. (lacrimal adj deficiency) | 0 |
| 1. (tear adj deficiency) | 1 |
| 1. (tear adj evaporation) | 1 |
| 1. (depleted adj tear adj secretion) | 0 |
| 1. (non-Sjögren adj keratoconjunctivitis adj sicca) | 0 |
| 1. (sjogren adj syndrome) | 6 |
| 1. (sjogren adj syndrome adj related adj dry adj eye adj disease) | 0 |
| 1. (sjogren adj syndrome adj related adj ocular adj surface adj disease | 0 |
| 1. (sjogren adj syndrome adj dry adj eye*) | 1 |
| **TOTAL** | 1,710 |

1. EMBASE (Ovid):, restrictions to: humans only

| **EMBASE (Ovid)** | |
| --- | --- |
| Content/construct | Nr of citations |
| 1. (ocular adj surface adj disease*) | 3,542 |
| 1. (OSD) | 581 |
| 1. (ocular adj surface adj condition*) | 93 |
| 1. (ocular adj surface adj disturbance*) | 10 |
| 1. (ocular adj surface adj syndrome*) | 5 |
| 1. (dry adj eye adj disease*) | 2,067 |
| 1. (dry adj eye adj condition*) | 163 |
| 1. (dry adj eye adj disturbance*) | 0 |
| 1. (dry adj eye adj syndrome*) | 2,004 |
| 1. (dry adj eye*) | 15,607 |
| 1. (DE) | 1,014,303 |
| 1. (DED) | 1,373 |
| 1. (keratoconjunctivitis adj sicca) | 2,667 |
| 1. (KCS) | 2112 |
| 1. (keratitis adj sicca) | 88 |
| 1. (KS) | 13,872 |
| 1. (dysfunctional adj tear adj syndrome*) | 48 |
| 1. (lacrimal adj keratoconjunctivitis) | 8 |
| 1. (evaporative adj tear adj deficiency) | 1 |
| 1. (aqueous adj tear adj deficiency) | 137 |
| 1. (lacrimal adj deficiency) | 8 |
| 1. (tear adj deficiency) | 235 |
| 1. (tear adj evaporation) | 184 |
| 1. (depleted adj tear adj secretion) | 0 |
| 1. (non-Sjögren adj keratoconjunctivitis adj sicca) | 0 |
| 1. (sjogren adj syndrome) | 3,962 |
| 1. (sjogren adj syndrome adj related adj dry adj eye adj disease) | 0 |
| 1. (sjogren adj syndrome adj related adj ocular adj surface adj disease | 0 |
| 1. (sjogren adj syndrome adj dry adj eye*) | 24 |
| **TOTAL** | 1,063,094 |

+ AND filter (1) applied:

(PRO integration or Clinical PRO application* or telePRO or automated PRO algorithm* or screening purpose* or PRO questionnaire* or Patient-reported outcome questionnaire* or Patient-reported symptom* or Patient-centred care or Patient self-report* or Self-report health or Self-rated health or Self-reported measure* of health or Health outcome* or Health communication* or Hospital performance evaluation* or Automated telephone survey system* or paper-based survey* or web-based survey* or web-based PRO platform* or web-based system* or PRO collection* or PRO measure* or PRO intervention* or PRO assessment intervention* or PRO data or PRO assessment* or Routine PRO assessment* or Routine PRO collection or Symptom assessment* or Symptom monitoring or Symptom data or Functional status or Electronic PRO assessment* or Electronic PRO system* or ePRO or ePRO* or ePRO system* or PRO system* or Generic PRO system* or PRO-based clinical alert system*).mp.

| **EMBASE (Ovid) + filter (1)** | |
| --- | --- |
| Content/construct | Nr of citations |
| 1. (ocular adj surface adj disease*) | 68 |
| 1. (OSD) | 9 |
| 1. (ocular adj surface adj condition*) | 1 |
| 1. (ocular adj surface adj disturbance*) | 0 |
| 1. (ocular adj surface adj syndrome*) | 0 |
| 1. (dry adj eye adj disease*) | 47 |
| 1. (dry adj eye adj condition*) | 1 |
| 1. (dry adj eye adj disturbance*) | 0 |
| 1. (dry adj eye adj syndrome*) | 24 |
| 1. (dry adj eye*) | 170 |
| 1. (DE) | 3,110 |
| 1. (DED) | 35 |
| 1. (keratoconjunctivitis adj sicca) | 22 |
| 1. (KCS) | 11 |
| 1. (keratitis adj sicca) | 1 |
| 1. (KS) | 39 |
| 1. (dysfunctional adj tear adj syndrome*) | 1 |
| 1. (lacrimal adj keratoconjunctivitis) | 0 |
| 1. (evaporative adj tear adj deficiency) | 0 |
| 1. (aqueous adj tear adj deficiency) | 0 |
| 1. (lacrimal adj deficiency) | 0 |
| 1. (tear adj deficiency) | 0 |
| 1. (tear adj evaporation) | 2 |
| 1. (depleted adj tear adj secretion) | 0 |
| 1. (non-Sjögren adj keratoconjunctivitis adj sicca) | 0 |
| 1. (sjogren adj syndrome) | 14 |
| 1. (sjogren adj syndrome adj related adj dry adj eye adj disease) | 0 |
| 1. (sjogren adj syndrome adj related adj ocular adj surface adj disease | 0 |
| 1. (sjogren adj syndrome adj dry adj eye*) | 1 |
| **TOTAL** | 3,556 |

1. PsycINFO (Ovid):, restrictions to: humans only

| **PsycINFO (Ovid)** | |
| --- | --- |
| Content/construct | Nr of citations |
| 1. (ocular adj surface adj disease*) | 17 |
| 1. (OSD) | 35 |
| 1. (ocular adj surface adj condition*) | 0 |
| 1. (ocular adj surface adj disturbance*) | 0 |
| 1. (ocular adj surface adj syndrome*) | 0 |
| 1. (dry adj eye adj disease*) | 9 |
| 1. (dry adj eye adj condition*) | 2 |
| 1. (dry adj eye adj disturbance*) | 0 |
| 1. (dry adj eye adj syndrome*) | 12 |
| 1. (dry adj eye*) | 96 |
| 1. (DE) | 99,418 |
| 1. (DED) | 33 |
| 1. (keratoconjunctivitis adj sicca) | 3 |
| 1. (KCS) | 97 |
| 1. (keratitis adj sicca) | 564 |
| 1. (KS) | 13,872 |
| 1. (dysfunctional adj tear adj syndrome*) | 0 |
| 1. (lacrimal adj keratoconjunctivitis) | 0 |
| 1. (evaporative adj tear adj deficiency) | 0 |
| 1. (aqueous adj tear adj deficiency) | 0 |
| 1. (lacrimal adj deficiency) | 0 |
| 1. (tear adj deficiency) | 0 |
| 1. (tear adj evaporation) | 1 |
| 1. (depleted adj tear adj secretion) | 0 |
| 1. (non-Sjögren adj keratoconjunctivitis adj sicca) | 0 |
| 1. (sjogren adj syndrome) | 83 |
| 1. (sjogren adj syndrome adj related adj dry adj eye adj disease) | 0 |
| 1. (sjogren adj syndrome adj related adj ocular adj surface adj disease | 0 |
| 1. (sjogren adj syndrome adj dry adj eye*) | 0 |
| **TOTAL** | 114,242 |

+ AND filter (2) applied:

(PRO integration or Clinical PRO application* or telePRO or automated PRO algorithm* or screening purpose* or PRO questionnaire* or Patient-reported outcome questionnaire* or Patient-reported symptom* or Patient-centred care or Patient self-report* or Self-report health or Self-rated health or Self-reported measure* of health or Health outcome* or Health communication* or Hospital performance evaluation* or Automated telephone survey system* or paper-based survey* or web-based survey* or web-based PRO platform* or web-based system* or PRO collection* or PRO measure* or PRO intervention* or PRO assessment intervention* or PRO data or PRO assessment* or Routine PRO assessment* or Routine PRO collection or Symptom assessment* or Symptom monitoring or Symptom data or Functional status or Electronic PRO assessment* or Electronic PRO system* or ePRO or ePRO* or ePRO system* or PRO system* or Generic PRO system* or PRO-based clinical alert system*).mp.

| **PsycINFO (Ovid) + filter (2)** | |
| --- | --- |
| Content/construct | Nr of citations |
| 1. (ocular adj surface adj disease*) | 2 |
| 1. (OSD) | 0 |
| 1. (ocular adj surface adj condition*) | 0 |
| 1. (ocular adj surface adj disturbance*) | 0 |
| 1. (ocular adj surface adj syndrome*) | 0 |
| 1. (dry adj eye adj disease*) | 1 |
| 1. (dry adj eye adj condition*) | 0 |
| 1. (dry adj eye adj disturbance*) | 0 |
| 1. (dry adj eye adj syndrome*) | 0 |
| 1. (dry adj eye*) | 4 |
| 1. (DE) | 480 |
| 1. (DED) | 2 |
| 1. (keratoconjunctivitis adj sicca) | 1 |
| 1. (KCS) | 2 |
| 1. (keratitis adj sicca) | 0 |
| 1. (KS) | 3 |
| 1. (dysfunctional adj tear adj syndrome*) | 0 |
| 1. (lacrimal adj keratoconjunctivitis) | 0 |
| 1. (evaporative adj tear adj deficiency) | 0 |
| 1. (aqueous adj tear adj deficiency) | 0 |
| 1. (lacrimal adj deficiency) | 0 |
| 1. (tear adj deficiency) | 0 |
| 1. (tear adj evaporation) | 0 |
| 1. (depleted adj tear adj secretion) | 0 |
| 1. (non-Sjögren adj keratoconjunctivitis adj sicca) | 0 |
| 1. (sjogren adj syndrome) | 0 |
| 1. (sjogren adj syndrome adj related adj dry adj eye adj disease) | 0 |
| 1. (sjogren adj syndrome adj related adj ocular adj surface adj disease | 0 |
| 1. (sjogren adj syndrome adj dry adj eye*) | 0 |
| **TOTAL** | 495 |

1. CINAHL Plus (EBSCO):, restrictions to: humans only

| **CINAHL Plus (EBSCO)** | |
| --- | --- |
| Content/construct | Nr of citations |
| 1. (ocular adj surface adj disease*) | 93 |
| 1. (OSD) | 44 |
| 1. (ocular adj surface adj condition*) | 19 |
| 1. (ocular adj surface adj disturbance*) | 9 |
| 1. (ocular adj surface adj syndrome*) | 25 |
| 1. (dry adj eye adj disease*) | 89 |
| 1. (dry adj eye adj condition*) | 14 |
| 1. (dry adj eye adj disturbance*) | 4 |
| 1. (dry adj eye adj syndrome*) | 21 |
| 1. (dry adj eye*) | 3 |
| 1. (DE) | 32,193 |
| 1. (DED) | 93 |
| 1. (keratoconjunctivitis adj sicca) | 33 |
| 1. (KCS) | 21 |
| 1. (keratitis adj sicca) | 4 |
| 1. (KS) | 410 |
| 1. (dysfunctional adj tear adj syndrome*) | 23 |
| 1. (lacrimal adj keratoconjunctivitis) | 10 |
| 1. (evaporative adj tear adj deficiency) | 5 |
| 1. (aqueous adj tear adj deficiency) | 5 |
| 1. (lacrimal adj deficiency) | 2 |
| 1. (tear adj deficiency) | 5 |
| 1. (tear adj evaporation) | 10 |
| 1. (depleted adj tear adj secretion) | 4 |
| 1. (non-Sjögren adj keratoconjunctivitis adj sicca) | 73 |
| 1. (sjogren adj syndrome) | 46 |
| 1. (sjogren adj syndrome adj related adj dry adj eye adj disease) | 168 |
| 1. (sjogren adj syndrome adj related adj ocular adj surface adj disease*) | 171 |
| 1. (sjogren adj syndrome adj dry adj eye*) | 21 |
| **TOTAL** | 33,417 |

+ AND filter (S3) applied:

""((((((((((((((((((((((((((((((Instrumentation or method* or Validation Studies or Comparative Study).mp. or psychometrics/ or psychometr*.mp. or clinimetr*.mp. or clinometr*.mp. or outcome assessment health care/ or outcome assessment*.ti,ab. or outcome measure*.mp. or observer variation/ or observer variation*.ti,ab. or Health Status Indicators/ or reproducibility of results/ or reproducib*.ti,ab. or discriminant analysis/ or reliab*.ti,ab. or unreliab*.ti,ab. or valid*.ti,ab. or coefficient of variation.ti,ab. or coefficient*.ti,ab. or homogeneity.ti,ab. or homogeneous.ti,ab. or internal consistency.ti,ab. or cronbach*.ti,ab.) and alpha*.ti,ab.) or item*.ti,ab.) and correlation*.ti,ab.) or selection*.ti,ab. or reduction*.ti,ab. or agreement.mp. or precision.mp. or imprecision.mp. or precise value*.mp. or test-retest.ti,ab. or test.ti,ab.) and retest.ti,ab.) or reliab*.ti,ab.) and test.ti,ab.) or retest.ti,ab. or stability.ti,ab. or interrater.ti,ab. or inter-rater.ti,ab. or intrarater.ti,ab. or intra-rater.ti,ab. or intertester.ti,ab. or inter-tester.ti,ab. or intratester.ti,ab. or intra-tester.ti,ab. or interobserver.ti,ab. or inter-observer.ti,ab. or intraobserver.ti,ab. or intra-observer.ti,ab. or intertechnician.ti,ab. or inter-technician.ti,ab. or intratechnician.ti,ab. or intra-technician.ti,ab. or interexaminer.ti,ab. or inter-examiner.ti,ab. or intraexaminer.ti,ab. or intra-examiner.ti,ab. or interassay.ti,ab. or inter-assay.ti,ab. or intraassay.ti,ab. or intra-assay.ti,ab. or interindividual.ti,ab. or inter-individual.ti,ab. or intraindividual.ti,ab. or intra-individual.ti,ab. or interparticipant.ti,ab. or inter-participant.ti,ab. or intraparticipant.ti,ab. or intra-participant.ti,ab. or kappa*.ti,ab. or kappa's.ti,ab. or repeatab*.mp. or replicab*.mp. or repeated.mp.) and measure*.mp.) or finding*.mp. or result*.mp. or test*.mp. or generaliza*.ti,ab. or generalisa*.ti,ab. or concordance.ti,ab. or intraclass.ti,ab.) and correlation*.ti,ab.) or discriminative.ti,ab. or known group.ti,ab. or factor analysis.ti,ab. or factor analyses.ti,ab. or factor structure.ti,ab. or factor structure.ti,ab. or dimension*.ti,ab. or subscale*.ti,ab. or multitrait.ti,ab.) and scaling.ti,ab. and analysis.ti,ab.) or analyses.ti,ab. or item discriminant.ti,ab. or interscale correlation*.ti,ab. or error.ti,ab. or errors.ti,ab. or individual variability.ti,ab. or interval variability.ti,ab. or rate variability.ti,ab. or variability.ti,ab.) and analysis.ti,ab.) or value*.ti,ab. or uncertainty.ti,ab.) and measurement.ti,ab.) or measuring.ti,ab. or standard error of measurement.ti,ab. or sensitiv*.ti,ab. or responsive*.ti,ab. or limit*.ti,ab.) and detection.ti,ab.) or minimal detectable concentration.ti,ab. or interpretab*.ti,ab. or minimal.ti,ab. or minimally.ti,ab. or clinical.ti,ab. or clinically.ti,ab.) and important.ti,ab.) or significant.ti,ab. or detectable.ti,ab.) and change.ti,ab.) or difference.ti,ab. or small*.ti,ab.) and real.ti,ab.) or detectable.ti,ab.) and change.ti,ab.) or difference.ti,ab. or meaningful change.ti,ab. or ceiling effect.ti,ab. or floor effect.ti,ab. or Item response model.ti,ab. or IRT.ti,ab. or Rasch.ti,ab. or Differential item functioning.ti,ab. or DIF.ti,ab. or computer adaptive testing.ti,ab. or item bank.ti,ab. or cross-cultural equivalence.ti,ab.)

| **CINAHL Plus (EBSCO)+ filter (S3)** | |
| --- | --- |
| Content/construct | Nr of citations |
| 1. (ocular adj surface adj disease*) | 9 (0) |
| 1. (OSD) | 0 |
| 1. (ocular adj surface adj condition*) | 2 (0) |
| 1. (ocular adj surface adj disturbance*) | 0 |
| 1. (ocular adj surface adj syndrome*) | 0 |
| 1. (dry adj eye adj disease*) | 0 |
| 1. (dry adj eye adj condition*) | 0 |
| 1. (dry adj eye adj disturbance*) | 0 |
| 1. (dry adj eye adj syndrome*) | 0 |
| 1. (dry adj eye*) | 0 |
| 1. (DE) | 17 (0) |
| 1. (DED) | 0 |
| 1. (keratoconjunctivitis adj sicca) | 0 |
| 1. (KCS) | 0 |
| 1. (keratitis adj sicca) | 0 |
| 1. (KS) | 0 |
| 1. (dysfunctional adj tear adj syndrome*) | 1 (0) |
| 1. (lacrimal adj keratoconjunctivitis) | 0 |
| 1. (evaporative adj tear adj deficiency) | 0 |
| 1. (aqueous adj tear adj deficiency) | 0 |
| 1. (lacrimal adj deficiency) | 0 |
| 1. (tear adj deficiency) | 0 |
| 1. (tear adj evaporation) | 0 |
| 1. (depleted adj tear adj secretion) | 0 |
| 1. (non-Sjögren adj keratoconjunctivitis adj sicca) | 0 |
| 1. (sjogren adj syndrome) | 0 |
| 1. (sjogren adj syndrome adj related adj dry adj eye adj disease) | 0 |
| 1. (sjogren adj syndrome adj related adj ocular adj surface adj disease*) | 0 |
| 1. (sjogren adj syndrome adj dry adj eye*) | 0 |
| **TOTAL** | 29 (0) |

+ AND Lee’s filter (S4) applied:

((PRO integration or Clinical PRO application* or telePRO or automated PRO algorithm* or screening purpose* or PRO questionnaire* or Patient-reported outcome questionnaire* or Patient-reported symptom* or Patient-centred care or Patient self-report* or Self-report health or Self-rated health or Self-reported measure* of health or Health outcome* or Health communication* or Hospital performance evaluation* or Automated telephone survey system* or paper-based survey* or web-based survey* or web-based PRO platform* or web-based system* or PRO collection* or PRO measure* or PRO intervention* or PRO assessment intervention* or PRO data or PRO assessment* or Routine PRO assessment* or Routine PRO collection or Symptom assessment* or Symptom monitoring or Symptom data or Functional status or Electronic PRO assessment* or Electronic PRO system* or ePRO or ePRO* or ePRO system* or PRO system* or Generic PRO system* or PRO-based clinical alert system*).mp. )""

| **CINAHL Plus (EBSCO)+ filter (S4)** | |
| --- | --- |
| Content/construct | Nr of citations |
| 1. (ocular adj surface adj disease*) | 0 |
| 1. (OSD) | 0 |
| 1. (ocular adj surface adj condition*) | 0 |
| 1. (ocular adj surface adj disturbance*) | 0 |
| 1. (ocular adj surface adj syndrome*) | 0 |
| 1. (dry adj eye adj disease*) | 0 |
| 1. (dry adj eye adj condition*) | 0 |
| 1. (dry adj eye adj disturbance*) | 0 |
| 1. (dry adj eye adj syndrome*) | 0 |
| 1. (dry adj eye*) | 0 |
| 1. (DE) | 0 |
| 1. (DED) | 0 |
| 1. (keratoconjunctivitis adj sicca) | 0 |
| 1. (KCS) | 0 |
| 1. (keratitis adj sicca) | 0 |
| 1. (KS) | 0 |
| 1. (dysfunctional adj tear adj syndrome*) | 0 |
| 1. (lacrimal adj keratoconjunctivitis) | 0 |
| 1. (evaporative adj tear adj deficiency) | 0 |
| 1. (aqueous adj tear adj deficiency) | 0 |
| 1. (lacrimal adj deficiency) | 0 |
| 1. (tear adj deficiency) | 0 |
| 1. (tear adj evaporation) | 0 |
| 1. (depleted adj tear adj secretion) | 0 |
| 1. (non-Sjögren adj keratoconjunctivitis adj sicca) | 0 |
| 1. (sjogren adj syndrome) | 0 |
| 1. (sjogren adj syndrome adj related adj dry adj eye adj disease) | 0 |
| 1. (sjogren adj syndrome adj related adj ocular adj surface adj disease*) | 0 |
| 1. (sjogren adj syndrome adj dry adj eye*) | 0 |
| **TOTAL** | 0 |

- Summary of records retrieved with corresponding filters (nr):

| 1. MEDLINE (Ovid) | 1,710 |
| --- | --- |
| 1. EMBASE (Ovid) | 3,556 |
| 1. PsycINFO (Ovid) filter 1 | 495 |
| 1. CINAHL Plus (EBSCO) | 0 |
| 1. Cochrane Library | 0 |
| **TOTAL** | 5,761 |
